# 

**Published:** 1904-01-02

**Authors:** 


### CONTENTS.

**Looking Back** - - - - - - - - - - - - - - 23

**The Liability of Voluntary Hospitals for Negligence** - - - - - - - - - - - - - 236

**Annotations** - - - - - - - - - - - - - - 237

**Notes and News** - - - - - - - - - - - - - 238

**Hospital Clinics and Medical Progress—**

1903 - - - - - - - - - - - - - - 239

What We Owe to Experiments on Animals - - - - - 242

PROGRESS IN DIABETES - - - - - - - - - - 244

DISEASES OF THE HEART AND CIRCULATION - - - - - 245

PROGRESS IN OPHTHALMOLOGY - - - - - - - 246

**New Appliances and Things Medical** - - - - 246

**Hospital Administration—**

The London Hospitals - - - - - - - - - - - 247

British Institutions for the Care of the Inebriate.—VI. - - 248

The Hospitals and Dispensaries of Burma - - - - - - 249

"The Doctor" - - - - - - - - - - - - 250

Construction Notes - - - - - - - - - - - 250

**The Student of Medicine** - - - - - - - - 250

*NURSING SECTION.*

**Notes on News from the Nursing World—**
A Splendid Memorial—Honours for Mr. Harold Boulton—The Scottish Branch of Queen Victoria's Jubilee Institute—The Japanese Army Nursing Service—The Midwives Board and Applications for Certificates—Nurses and Public Worship—State Registration—Nurses' Home at the Cancer Hospital—Improvements at Royal Hants County Hospital—The Nurses of Bristol Children's Hospital—The Matron of Brechin Infirmary—Nursing in Northern Workhouse Infirmaries—Painful Charge against a Nurse—The Nurses of Greenwich Workhouse Infirmary—Proposed New Departure at Truro—Amateur Nurses and the Finsen Lamp—Our Christmas Distribution - - - - - - - - - 187-190
**The Nursing Outlook** - - - - - - - - - 191
**Lectures upon the Nursing of Mental Diseases** 192
**Christmas in the Hospitals** - - - - - - - 193
**Everybody's Opinion** - - - - - - - - - 196
**Death in Our Ranks** - - - - - - - - - 196
**Appointments** - - - - - - - - - - - 196
**Presentations** - - - - - - - - - - - 196
**Echoes from the Outside World** - - - - - - 197
**Notes and Queries** - - - - - - - - - 198
**For Reading to the Sick** - - - - - - - - 198

